# Genenames.org: the HGNC and VGNC resources in 2019

**DOI:** 10.1093/nar/gky930

**Published:** 2018-10-10

**Authors:** Bryony Braschi, Paul Denny, Kristian Gray, Tamsin Jones, Ruth Seal, Susan Tweedie, Bethan Yates, Elspeth Bruford

**Affiliations:** HUGO Gene Nomenclature Committee, European Molecular Biology Laboratory, European Bioinformatics Institute, Wellcome Genome Campus, Hinxton, Cambridgeshire CB10 1SD, UK

## Abstract

The HUGO Gene Nomenclature Committee (HGNC) based at EMBL’s European Bioinformatics Institute (EMBL-EBI) assigns unique symbols and names to human genes. There are over 40 000 approved gene symbols in our current database of which over 19 000 are for protein-coding genes. The Vertebrate Gene Nomenclature Committee (VGNC) was established in 2016 to assign standardized nomenclature in line with human for vertebrate species that lack their own nomenclature committees. The VGNC initially assigned nomenclature for over 15000 protein-coding genes in chimpanzee. We have extended this process to other vertebrate species, naming over 14000 protein-coding genes in cow and dog and over 13 000 in horse to date. Our HGNC website https://www.genenames.org has undergone a major design update, simplifying the homepage to provide easy access to our search tools and making the site more mobile friendly. Our gene families pages are now known as ‘gene groups’ and have increased in number to over 1200, with nearly half of all named genes currently assigned to at least one gene group. This article provides an overview of our online data and resources, focusing on our work over the last two years.

## INTRODUCTION

The HUGO Gene Nomenclature Committee (HGNC) is the only internationally recognized authority tasked with assigning unique and informative gene symbols and names to human genes. All HGNC public data, tools and accompanying help documentation can be accessed via the https://www.genenames.org website.

Standardized HGNC approved nomenclature is used in publications and biomedical databases to remove ambiguity and facilitate communication between researchers worldwide. HGNC symbols are displayed in all major databases containing human gene and protein data including Ensembl (1), NCBI Gene (2), UniProt (3), GeneCards (4) and the UCSC genome browser (5), as well as resources focused on human disease and phenotypes such as Decipher (6), OMIM (7), Locus Reference Genomics (LRG) (8), ClinVar (9) and GeneTests (10).

We established the Vertebrate Gene Nomenclature Committee (VGNC) in 2016 to assign standardized nomenclature in line with human for vertebrate species that lack their own nomenclature committees (11). We are currently naming genes in chimpanzee, dog, cow and horse, and all of this data can be found on the dedicated VGNC website https://vertebrate.genenames.org.

## DATA

**Figure 1.**
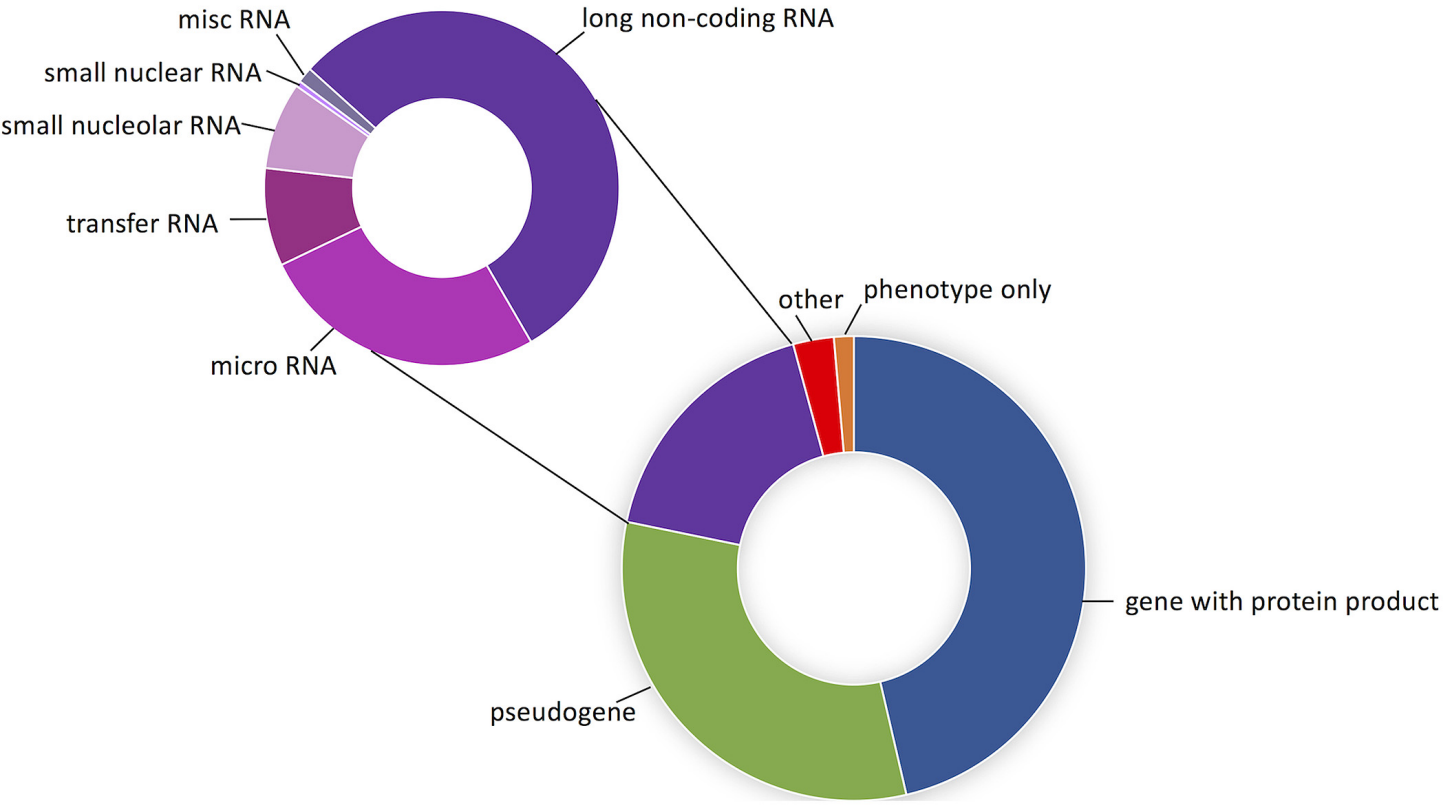
The proportion of HGNC gene symbols annotated with each locus type. The main doughnut chart shows the proportions of major locus groups. The purple region represents genes annotated with the non-protein-coding RNA locus group; the smaller chart shows the proportion of RNA genes annotated with each RNA-specific locus type. The ‘misc RNA’ category groups together RNA locus types that represent a small number of genes. A full list of locus types, along with total numbers of approved symbols for each category, can be viewed at our Statistics and Downloads facility (www.genenames.org/stats).

As of September 2018, we have 41 439 approved gene symbols within our HGNC database, of which 19 194 are for protein-coding genes (Figure 1). Although the number of protein-coding genes is plateauing, nevertheless changes in locus type and new gene annotations have increased our total by nearly 200 in the last two years. We have been continuing to standardise and simplify human gene names prior to their transferral to other species as part of our ongoing VGNC project. This has included removing reference to species from gene names originally assigned based on an orthologous gene and removing reference to human phenotypes where possible. Just over 600 edits have been made to human protein-coding gene names (but not symbols) since September 2017.

## HUMAN NOMENCLATURE UPDATES

HGNC have three main types of placeholder symbols: C#orf#s are assigned to predicted genes designated by the chromosome of origin, the letters ‘orf’ for open reading frame and an iterative number. KIAA#s are approved for genes identified by the Kazusa cDNA sequencing project (12) when no other information is known about the gene. Finally, the FAM# root is used to group together a set of genes that are related based on sequence similarity, but cannot be described by function or conserved domains. HGNC policy has always been to view placeholder symbols as temporary assignments that should be updated when the function of a protein encoded by a gene is identified.

As of September 2018, we have only 619 approved protein-coding genes with placeholder symbols: 341 C#orf#s, 43 KIAA#s and 235 FAM#s. This is a 20% reduction in the number of placeholder symbols during the last two years. We have been able to update placeholder symbols based on publications defining the function of their encoded proteins and/or on gene family membership. Where little human functional data is available, we use information such as orthology with a characterized gene in another species, or the presence of conserved domains within an encoded protein. In many cases a placeholder symbol has been updated based on a combination of these factors.

Recent updates have included four C#orf#s that were identified by the Chlamydomonas Flagellar Proteome Project (http://chlamyfp.org/index.php) as the human orthologs of Flagella Associated Protein genes in Chlamydomonas. These human genes were assigned a CFAP# root (cilia and flagella associated protein) and a number in line with their associated FAP# ortholog (e.g. the human ortholog of Chlamydomonas FAP410 has been assigned as *CFAP410*). In the few cases where these genes were published they were linked with human ciliopathies, highlighting that the CFAP# root is useful and appropriate.

In a minority of cases where the community working on a gene is very keen to keep a placeholder symbol that has become entrenched in the literature, we may be able to consider this on a case-by-case basis. We recently wrote to researchers proposing a nomenclature update for the family of paralogs currently approved using the placeholder root symbol FAM20#. These genes have been highly published on (>100 papers) and it was difficult to reach a consensus on what a new symbol for them should be, given the disparate functions of the encoded proteins. The situation was also complicated by *FAM20C* being published on using the alias symbol G-CK (Golgi casein kinase). We could not approve this symbol, as it contains a hyphen, and without it, clashes with the approved gene symbol for an unrelated gene, *GCK* (glucokinase) so could not be approved. The majority view was that the community wanted to keep FAM20# as a root symbol. Therefore, we have retained the FAM20# symbols and updated the gene names to reflect function: *FAM20A* (FAM20A, Golgi associated secretory pathway pseudokinase), *FAM20B* (FAM20B, glycosaminoglycan xylosylkinase) and *FAM20C* (FAM20C, golgi associated secretory pathway kinase).

We are currently working towards minimizing future gene symbol changes and as members of the TGMI (Transforming Genetic Medicine Initiative) (http://www.thetgmi.org/). We are prioritizing gene symbol stabilization for genes associated with human phenotypes. We are working towards identifying an initial set of gene symbols to designate as long-term stabilized.

### Non-coding RNAs and pseudogenes

In addition to our work naming protein-coding genes, the HGNC also names small and long non-coding RNA genes and pseudogenes (Figure 1). We work directly with specialist advisors to name small non-coding RNA genes and in 2017, following discussions with gene annotators at the RefSeq project (13) and with our advisor Dr Andrew Pierce we named a set of putative ribosomal RNA genes that are on the human reference genome GRCh38 but that are outside of the unsequenced large ribosomal clusters where functional ribosomal RNA genes are known to reside (see our gene group: ‘Ribosomal 45S rRNA genes outside of clusters’).

The long non-coding RNA (lncRNA) field is rapidly expanding and the HGNC aims to help this relatively new community by approving symbols for those genes that are discussed the most. We have named 659 new lncRNA genes since January 2017, representing a 16% increase in the total number of named lncRNA genes. Our highest priority for naming is lncRNA symbol requests received from authors, followed by published lncRNA genes. Where possible we approve the exact symbol that has been published, e.g. *TBILA* (14), but if the symbol does not follow our guidelines then we contact the groups involved to discuss a suitable alternative, e.g. the gene published as *THOR* (15) has been approved as *THORLNC*. After published lncRNAs, our next priority for naming is lncRNA genes that have been consistently annotated by the Gencode and RefSeq projects. We name these based on genomic location, including using the root symbol LINC# for ‘long intergenic non-protein coding’ lncRNA genes. We have recently produced a test dataset for an initial exploration into the possibility of using machine learning to name intergenic lncRNA genes that meet certain defined criteria. We will report back on this project in future publications. Complex lncRNA gene models and those that overlap or are divergent to protein-coding genes will always require manual naming.

Much of our pseudogene naming has recently been in the course of work on specific gene groups e.g. *SELENOKP1* was named following the approval of gene nomenclature for the protein-coding parent, *SELENOK*. We are prioritizing the naming of pseudogenes based on type, starting with those that are mainly unprocessed and consistently annotated. Some of these are subsequently found to be orthologous to protein coding genes in other species when we look across species as part of the VGNC project, for example we named *VAMP9P* based on its orthology to the published protein-coding rodent gene Vamp9 (16).

## GENE GROUPS

We manually curate genes into groups based on shared characteristics such as homology, associated phenotype and encoded protein function. These were previously referred to as ‘Gene Families’ (17), but we now call these ‘Gene Groups’ on our new website, to better reflect that these diverse groupings are not limited to being based on sequence homology alone. Illustrating this diversity, some of the recent additions to our Gene Groups are: nineteen new groups covering the complexes of the major and minor spliceosomes; the ‘Complement system’, subdivided into ‘Complement system regulators and receptors’ and ‘Complement system activation components’; and ‘Small NF90 (ILF3) associated RNAs’ as a new group of ‘non-coding RNAs’.

We try to name related genes using a shared root symbol but this is not always possible, as genes may already be well published using another symbol; hence our ‘Gene Groups’ provide a mechanism to find and view related genes with disparate nomenclature, e.g. the human chromatin accessibility complex (CHRAC) comprises *CHRAC1, POLE3, BAZ1A* and *SMARCA5*, only the first of which is named using the root symbol that designates it as a subunit of this complex.

The number of HGNC Gene Groups reached 1250 in August 2018—an increase of 25% in the last 2 years. Nearly half (47%) of all named genes are currently assigned to at least one gene group, and over two thirds (68%) of protein coding genes are in at least one group. Historically, enzymes have been underrepresented in our gene groups—20% of the 6152 protein coding genes not yet assigned to a group are named as enzymes or as containing enzyme domains. We are gradually addressing this and recently added 11 new groups of glycoside hydrolases. These broadly follow the Glycoside hydrolase (GH) families defined in *CAZypedia*, (http://www.cazypedia.org/index.php/Glycoside_Hydrolase_Families accessed 31 July 2018). The pre-existing lysozyme, mannosidase and chitinase groups are now listed as subgroups of the ‘Glycoside hydrolases’ making it is possible to view the complete hierarchy and download all 92 human glycoside hydrolase genes.

We continue to maintain links to related Gene Groups of *Drosophila* genes curated by FlyBase (18). Almost a third (274/881) of FlyBase Groups are mapped to an HGNC gene group (FB2018_03, released 19 June 2018).

## VGNC

To ensure that genes in model organisms are named in line with their human homologs, we work closely with the five active vertebrate nomenclature committees: MGNC (mouse) (19), RGNC (rat) (20), CGNC (chicken) (21), XNC (*Xenopus*) (22) and the ZNC (zebrafish) (23). The VGNC assigns nomenclature for vertebrate species that lack their own nomenclature committees, prioritizing species based on the quality of their genome assemblies, value as model organisms and interest from the communities working on them.

**Figure 2.**
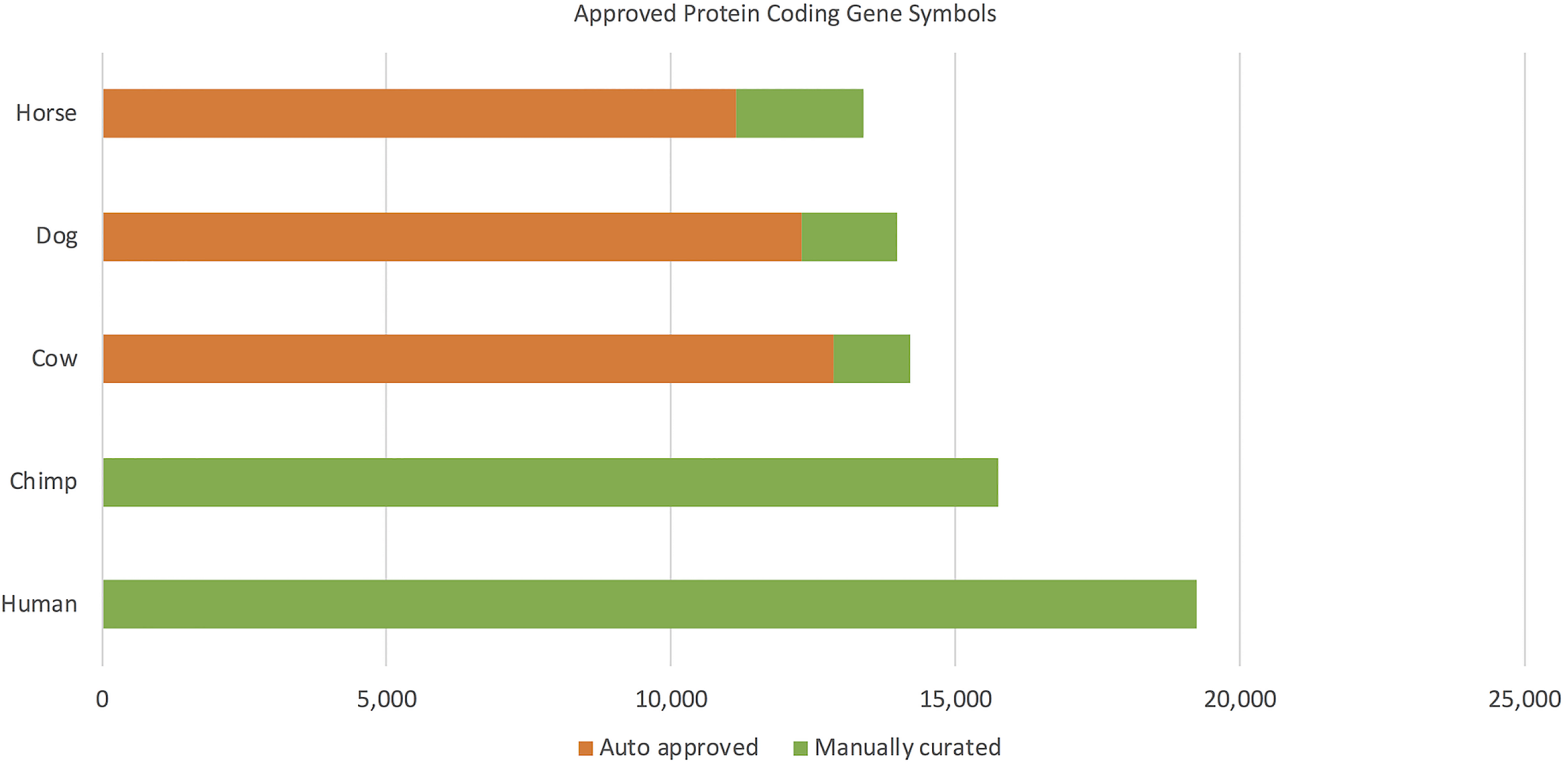
Genes with approved nomenclature for VGNC species that have been auto approved or manually curated.

VGNC began naming chimpanzee genes in 2016 and over the past two years the total number of protein-coding chimpanzee genes has increased by 1250 to 15 752 due to continued manual curation efforts (Figure 2). In the same period we have extended the process to assign nomenclature for protein-coding genes in cow (14 181), dog (13 983) and horse (13 378). The NCBI Gene and Ensembl databases now display approved VGNC nomenclature for all relevant entries, along with reciprocal links to the symbol reports on the VGNC site (https://vertebrate.genenames.org/).

Our naming strategy uses a subset of data from our HCOP (HGNC Comparison of Orthology Predictions) tool (24). This tool combines orthology assertions made by fourteen resources into a single resource (https://www.genenames.org/cgi-bin/hcop). HCOP has recently been updated to include data for two new species, cat and fission yeast. The addition of *Schizosaccharomyces pombe* has led to the inclusion of nomenclature and orthology data from PomBase (25) as well as orthology data from Ensembl Genomes (26).

For each vertebrate species, a high confidence set of 1:1 ortholog predictions from the HCOP tool is produced. The resulting vertebrate gene sets are then assigned the same gene nomenclature as the human orthologs using an automated pipeline. We are now focusing on naming vertebrate genes that have lower confidence orthology predictions to human genes. These require careful manual review across multiple species which may include steps such as assessing synteny, comparing phylogenies available from a selection of HCOP resources, and reviewing the literature. For example, we are increasingly encountering examples of species-specific duplications that mean we must take care when naming orthologs across vertebrates, as illustrated by a species-specific duplication post-speciation event that has resulted in two copies of the ancestral *MMP23* gene in human: *MMP23A* (matrix metallopeptidase 23A (pseudogene)) and *MMP23B* (matrix metallopeptidase 23B). Therefore, the vertebrate genes have simply been approved as *MMP23* (matrix metallopeptidase 23). A subset of these belong to complex gene families which may necessitate consultation with specialist advisors before approval. Currently we are working with experts on the complex cytochrome P450 and olfactory receptor families across vertebrates.

## NEW WEBSITE

Our last major update of www.genenames.org was released in May 2011 to provide a public access portal to our database, built using the Drupal content management system and Perl common gateway interface. This year we designed a new version of our website that has been developed utilising user experience (UX) testing and using Jekyll, Bootstrap and the AngularJS Framework to bring the static and dynamic content into the same system. These newer technologies have enabled us to create a mobile first website with reusable components. When viewed on a mobile device, navigation tools and menus are collapsed for a simpler display, whilst still retaining the functionality of the full site.

**Figure 3.**
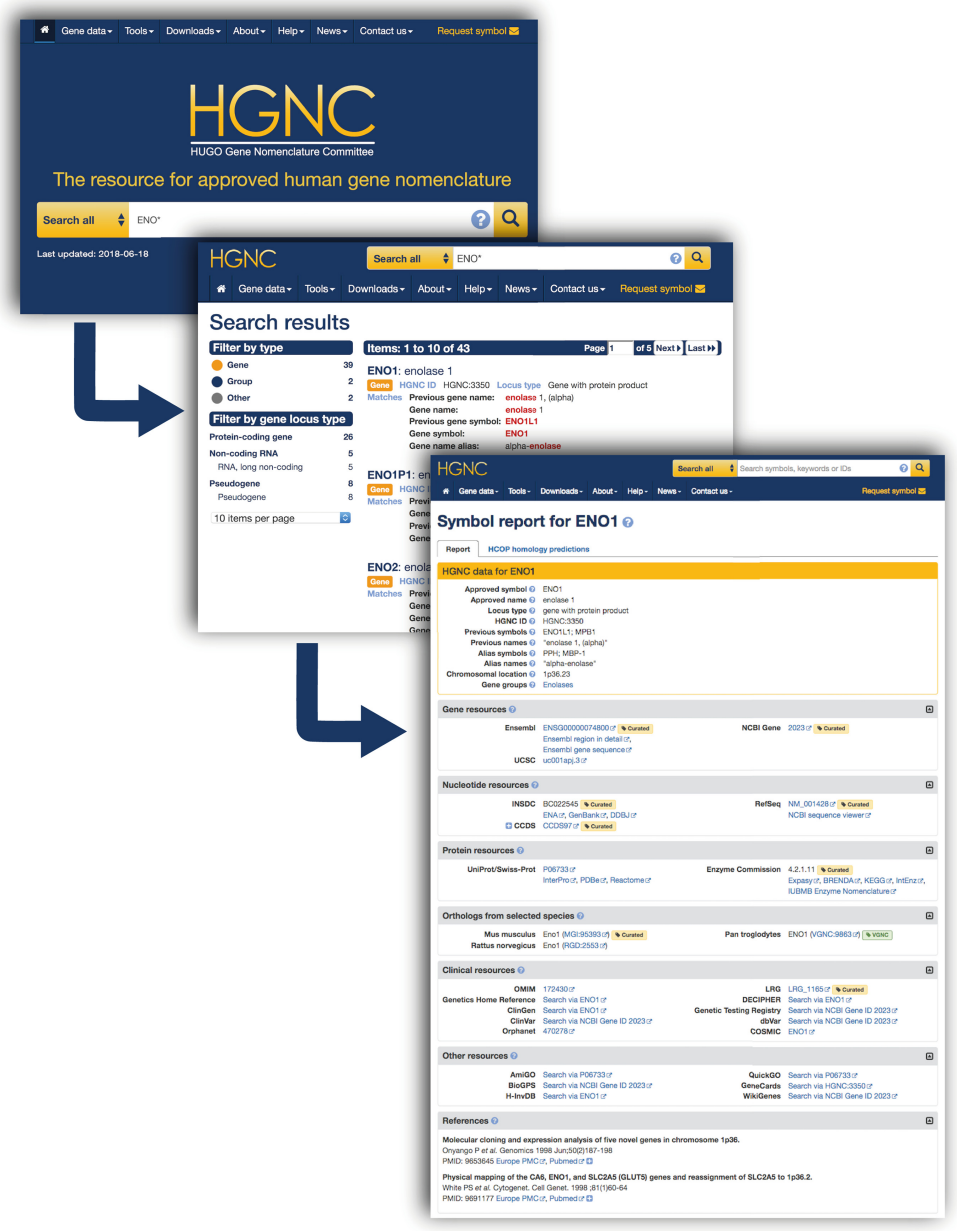
Screenshots to show the homepage, an example search results page and a gene symbol report page from the HGNC website (https://www.genenames.org).

The new homepage has been greatly simplified to highlight our site search (Figure 3). The search box includes a drop-down selection to allow the choice of searching all site content, or restricting searches to gene symbol reports, gene groups, or other text-based pages. UX testing highlighted that users want to know when the HGNC resource was last updated, so this has been added below the search box. We have included colour coding in the search results page to allow users to distinguish between the types of page their search has found. User testing prompted us to display the locus type for a gene next to the HGNC ID, as this is a key piece of data that it is useful to highlight. Search result filters are shown on the left of the page. When viewing on a mobile device these filters are located within a menu that can be activated via a button. Users can filter results by page type, locus group and/or locus type.

## GENE SYMBOL REPORTS

Our gene symbol reports will still be familiar to users, retaining the core HGNC data section at the top of each page in a golden panel box. A luggage tag labelled ‘curated’ shows that the associated data has been curated by us. The absence of this tag logically denotes that the data have been imported from an external resource. The external cross references section from the previous design has been broken down into collapsible labelled panels. These eight panels have been ordered based on usage statistics for the most used cross references. In addition, we now include a tab to display HCOP (HGNC Comparison of Orthology Predictions) results for a gene.

The new ‘Orthologs from selected species’ panel replaces the homologs panel from our previous design. We have expanded on the data included here by displaying and linking to ortholog data from our sister VGNC site (https://vertebrate.genenames.org) into this panel, denoted with green luggage tags labelled ‘VGNC’. We have added the following new links: ClinVar, ClinGen (27) and dbVar (28) have been added to the ‘Clinical resources’ panel and AmiGO (29) links have been added to the ‘Other resources’ section.

Our previous gene symbol reports contained mappings to a single UniProt/SwissProt entry per gene. In the new gene symbol report, we now provide the option to view all SwissProt accessions that UniProt have cross referenced to a given gene. This also applies to CCDS (30) and NCBI RefSeq accessions if more than one is mapped to a gene.

## GENE GROUP REPORTS

New features include the ability to download data about genes contained in a group or in all subgroups, available in tab delimited text, CSV or JSON format. We have also updated our hierarchy map viewing tool for our gene groups pages. Users can click directly on a box representing a group or subgroup to load the appropriate reports, or click a toggle switch to enter rearrangement mode and create customized screenshots.

## UPDATES TO TOOLS AND DOWNLOADS

Our popular HCOP and Multi-symbol checker tools on the beta site will look familiar to users but have been updated to be more mobile friendly. Our Multi-symbol checker tool allows users to input a list of gene symbols to check if they are currently HGNC approved, and the results page now allows the user to sort and filter data and customize the way it is displayed, as well as download the full results table in a comma separated file.

We have also updated our download tools—a RESTful API, a custom downloads tool, a BioMart server and download file links in gene group reports and on the ‘Statistics and Downloads’ page—to work better on mobile devices. For example, the chromosome image map in the downloads section has been replaced with a drop-down selection box when viewed on a mobile, as the image map did not scale well on smaller screens. Users can still view and download data by chromosome.

## FUTURE DIRECTIONS

### HGNC

We will continue to systematically approve nomenclature for all newly identified human protein coding genes, as well as consistently annotated and published non-coding RNA genes and pseudogenes. We will also continue to update temporary placeholder symbols to more appropriate function-based nomenclature whenever possible, in line with our ongoing aim of stabilizing as many protein coding gene symbols as possible. We will also continue to create new gene group pages, often based on publications.

Following on from the release of our newly designed website we plan to implement a new advanced HGNC search, allowing users to easily build more complex search queries. We also plan to expand our RESTful API in the downloads section of our site to include gene groups.

An upcoming feature for our gene symbol reports will be the addition of HGNC curators’ comments. These will not feature on every report, but will be used to highlight issues that could cause confusion to users. For example, the new comments section may be used to highlight controversy over the function of a gene product that may affect its nomenclature or to flag genes where annotation groups disagree on the locus type.

### VGNC

We will continue to standardize and simplify human nomenclature to make it suitable for transfer to orthologs across vertebrate species, and will continue both automated gene name transfer and manual curation of vertebrate orthologs of human genes. In line with our human gene groups data we will extend our coverage across selected vertebrate species for certain groups. We also plan to integrate some of the improvements made to our HGNC site into the VGNC site and to implement a RESTful API in the downloads section of our site for VGNC data.

We plan to improve our curator tools to facilitate orthologous gene curation in several species at once. We will be developing a semi-automated pipeline involving the comparison of phylogenetic trees to aid in the curation of more complex homology relationships across a wider range of vertebrates.

Please email us if you have expertise in a particular species or gene family you could help us to name: vgnc@genenames.org.

We will continue to add new species to VGNC based on the quality of genome assembly and annotations, perceived importance as a model for humans and demand from the research community.
